# Feasibility of Observing Cerebrovascular Disease Phenotypes with Smartphone Monitoring: Study Design Considerations for Real-World Studies

**DOI:** 10.3390/s24113595

**Published:** 2024-06-02

**Authors:** Stephanie J. Zawada, Ali Ganjizadeh, Clint E. Hagen, Bart M. Demaerschalk, Bradley J. Erickson

**Affiliations:** 1 Mayo Clinic College of Medicine and Science, 5777 E. Mayo Boulevard, Scottsdale, AZ 85054, USA; 2 Mayo Clinic AI Laboratory, 200 1st Street SW, Rochester, MN 55902, USA; ganjizadeh.ali@mayo.edu (A.G.); bje@mayo.edu (B.J.E.); 3 Mayo Clinic Division of Biomedical Statistics and Informatics, 200 1st Street SW, Rochester, MN 55902, USA; Hagen.Clinton@mayo.edu; 4 Mayo Clinic Center for Digital Health, 5777 E. Mayo Boulevard, Scottsdale, AZ 85054, USA; demaerschalk.bart@mayo.edu

**Keywords:** real-world data, smartphone sensors, health outcomes, remote patient monitoring, post-stroke depression, cerebrovascular disease

## Abstract

Accelerated by the adoption of remote monitoring during the COVID-19 pandemic, interest in using digitally captured behavioral data to predict patient outcomes has grown; however, it is unclear how feasible digital phenotyping studies may be in patients with recent ischemic stroke or transient ischemic attack. In this perspective, we present participant feedback and relevant smartphone data metrics suggesting that digital phenotyping of post-stroke depression is feasible. Additionally, we proffer thoughtful considerations for designing feasible real-world study protocols tracking cerebrovascular dysfunction with smartphone sensors.

## 1. Introduction

With stroke prevalence on the rise in young and middle-aged adults, the burden of cerebrovascular dysfunction poses an imminent challenge to both the healthcare system and global economy [1]. As cerebrovascular diseases (CeVDs), like transient ischemic attack (TIA) and stroke, increase the risk of subsequent CeVD diagnoses, especially stroke and dementia, the need to identify CeVD patients most at risk of worsening outcomes is critical [2]. In the U.S. alone, annual hospitalization and rehabilitation costs for stroke are projected to reach $184 billion by 2030, with some estimates accounting for indirect costs—like home care, follow-up consults, prescriptions, and lost earnings—to exceed $1 trillion by 2050 [3,4]. For post-stroke patients at risk of dementia, early detection of cognitive impairment (CI) could contribute to savings of $7 trillion in treatment and care costs [5].

Post-stroke depression (PSD) is one behavioral biomarker indicative of poor cognitive, rehabilitation, and survival outcomes [6]. Numerous instruments to assess PSD exist, though their translation into routine clinical practice is limited by little consistency with regard to validity and reliability [7,8]; however, emerging research suggests that PSD may be characterized by changes in the brain’s reward system [9]. PSD is also linked with post-stroke dementia and CI. In some PSD patients, treatment for depression simultaneously improves post-stroke dementia and CI [10]. Thus, identifying patients after hospitalization for stroke or TIA who would benefit from treatment for depression holds promise for subsequent stroke prevention and long-term cognitive outcomes.

The multifactorial etiology of PSD and heterogenous nature of post-stroke CI highlight the need for personalized care. Coupled with the development of low-cost wearable and minimally intrusive sensor monitoring, digital sensor metrics have the potential to generate novel phenotypes reflective of accumulating pathology in real-world settings before a diagnosis [11]. Ecological momentary assessment (EMA) methods have been validated for PSD risk prediction, sampling participant mood symptoms via DSM-IV criteria surveys delivered through a personal digital assistant tool over a one-week timeframe [12,13,14]; however, as these studies were conducted before the widespread adoption of smartphones and digital health during the COVID-19 pandemic, none involved continuous sensor monitoring. Jean et al. found that depressed mood was less severe in participants with more social interactions [12]. Sibon et al. found that the percentage of participants with elevated depression scores was the same at baseline and 3 months, but depression profile consistency across participants was low [14]. Since then, sensor-based technologies, such as accelerometers, have been applied to investigate the predictive potential of behavioral monitoring outside of clinical settings [15]. Using one week of accelerometer monitoring, a large population cohort study of adults in the UK Biobank found that accelerometer sensors may capture significant aberrations in hourly movement patterns linked with depression, such as changes in sleep and sedentary behavior, before a CeVD diagnosis [16]. In the Rotterdam Study longitudinal population cohort, significant deviations in basic and instrumental activities of daily living (BADL and IADL) and mood, as captured by the Mini-Mental State Examination (MMSE), may emerge within the 7 years leading to a stroke [17].

Beyond the predictive potential of mood tracking after a CeVD diagnosis, sensors capturing continuous data samples may generate novel outcome measures for clinical trials and phase IV (post-market) surveillance of drugs and devices [18]. As the use of hardware- and software-based digital health technologies (DHTs) continues to rise, questions about their application as real-world evidence (RWE) for the safety and efficacy monitoring of medical products have emerged [19].

Our protocol feasibility study builds on evidence and questions outlined above, using a smartphone app that blends active data collection via weekly surveys and passive data collection via GPS and accelerometer sensors over a 4-week period. Another novel feature of our study is the inclusion of post-TIA patients, for whom no EMA studies have been published and who are at a heightened risk of subsequent stroke and future dementia. Considering that post-ischemic stroke and TIA patients often experience temporary cognitive and mood changes as well as physical impairment, it is crucial to involve such users in protocol evaluation to ensure real-world studies are feasible and yield clinically meaningful conclusions. Furthermore, to enhance technology acceptance and ensure high-quality data collection, feasibility studies are helpful for the introduction of novel monitoring sensors, particularly those in bring-your-own-device studies [20]. The application of mixed methods is ideal to study factors associated with smartphone app use for clinical research [21].

In this perspective, we present a feasibility study for a smartphone sensor-based digital phenotyping protocol to capture behavior after ischemic stroke and TIA. Our feasibility assessment includes participant usability interviews and data quality metrics. We contextualize the results of our assessment in the context of study design considerations and future directions for digital phenotyping applied to CeVD. 

## 2. Methods

### 2.1. Protocol and Scientific Rationale

To evaluate the feasibility of a digital phenotyping protocol for post-TIA and ischemic stroke patients, our study design required participants to download the Beiwe digital phenotyping app on an iOS or Android smartphone. The Beiwe app is the participant-facing component of “an open-source, end-to-end encrypted digital phenotyping platform… [that includes] HIPAA-compliant cloud-based data storage” [22]. Participants were required to complete a validated depression questionnaire at baseline and once every 7 days for 4 weeks. Passive data collection was streamed continuously over the study period, with participants enabling location sharing (GPS) on their phones. At the study’s end, participants completed an individual exit interview to elicit user issues. The feasibility assessment metrics for primary active and passive data features included in the study, along with their future clinical application in a pilot study, are described in Table 1.

The intra-individual metrics derived from smartphone sensors represent potential digital biomarkers of PSD behavior or, when considered in combination, digital phenotypes of PSD; however, these have yet to be validated in post-stroke/TIA patients. 

Each hypothesis for future clinical use is supported by scientific rationale. For instance, with relevant mood surveys, summary scores assessing depressive symptoms increase as depression severity increases [23]. Also, prior research shows that depressed patients exhibit slower response times [24]. Reduced physical activity and more sedentary behavior has been well-established in depressed patients [25]. Social isolation has also been linked to PSD and long-term disability from stroke [26]. Evaluating summary statistics—and data missingness—for these passive and active features in our cohort is necessary to inform the development of a feasible digital phenotyping protocol.

### 2.2. Protocol Feasibility Study Design 

To evaluate the feasibility of our protocol, we used the theoretical framework “medical device technology development process” outlined by Shah et al. [27]. This framework consists of 5 stages in which end-users are involved in the development of a medical technology: (1) concept stage (idea generation and concept development), (2) design stage (device (re-)design and prototype development), (3) testing and trials stage (prototype testing in-house and trials in the real field), (4) production stage (device production based on business and commercial rationale), and (5) deployment stage (product launch and use in the market and post-deployment user feedback) [27]. We adapted the framework for our feasibility study, using an iterative process bridging stages (2) and (3) to optimize the protocol for our target study population: post-stroke/TIA patients. 

First, we applied stage 3, using issues communicated in-person or via email by participants who were lost to follow-up or withdrew from the study. Based on their feedback, the protocol was redesigned in an iterative fashion (stage 2), after which we re-applied stage 3, with the modified protocol, to continue the feasibility analysis.

Drawing on qualitative and quantitative feasibility data, we outlined fundamental considerations, linked to common characteristics of big data (volume, velocity, variety, veracity, and value), to help researchers optimize their real-world study designs, especially those relying on smartphone sensors [28].

### 2.3. Sample Population

Participants admitted to the Mayo Clinic Hospital in Scottsdale, Arizona, were included in the study, considering the following eligibility criteria: presenting with TIA or ischemic stroke symptoms, 18 years of age or older, and the ability to give verbal and/or written informed consent. Participants with a prior dementia diagnosis were excluded.

A convenience sampling strategy was used to recruit participants. Considering prototype study guidance outlined by Alroobaea and Mayhew, we aimed to enroll 16 participants in the feasibility study [29]. 

Printed and electronic flyers outlining necessary information about the study’s relevance, timeline, and participation requirements were provided directly to participants screened as eligible for the study. Participants were recruited with the assistance of the Cerebrovascular Diseases Division Chair, 1 attending neurologist, and 1 vascular stroke fellow. Study registration occurred online or in person, based on participant prerogative. Mayo Clinic IRB approved this study. Written informed consent was obtained prior to study participation.

### 2.4. Data Collection and Analysis

Baseline and demographic data were obtained from electronic health records (EHRs). Smartphone data was streamed from participant smartphones and stored on HIPAA-compliant Beiwe servers. For each participant, summary metrics for feasibility assessment outlined in Table 1 were computed.

Each participant who completed the study participated in a 10-to-15 min exit interview to assess satisfaction with the app. Participants were asked if (1) they encountered any issues on their phone since downloading the app and (2) if they had any feedback regarding the study. Responses were transcribed and reported. Participants who withdrew from the study were contacted for feedback via email. Troubleshooting requests via email or phone were noted and reported.

## 3. Results

**Figure 1:**
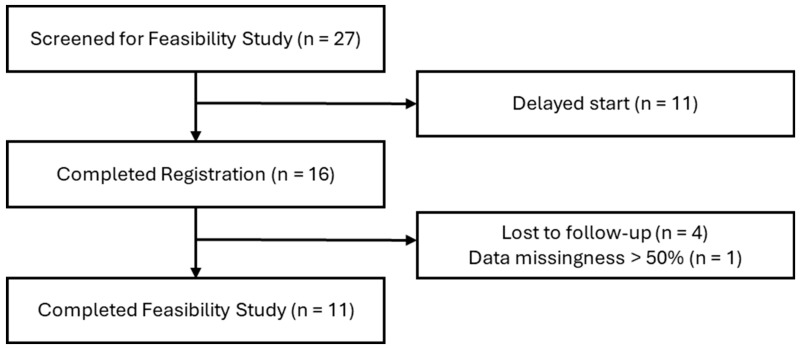
Participant Enrollment Flowchart.

From September 2023 to March 2024, 27 TIA and IS participants were screened for inclusion in the feasibility study (Figure 1). Eleven participants were excluded due to participant scheduling delays, and 16 participants completed the app registration process, which included downloading the app and configuring a password. Of the 16 who registered, 1 participant was excluded from analysis due to data incompleteness, and 4 participants were lost to follow-up. Among those lost to follow-up, two completed 1 week of data streaming and surveys after hospital discharge, 1 lacked an eligible phone for outside of the hospital, and 1 shared a smartphone with a spouse.

While the majority of the participants were TIA patients (75%), both ischemic stroke and TIA patients were mostly male (75% and 58.3%, respectively) and White (75% and 75%, respectively), and all provided a phone number (Table 2). TIA patients were older (mean age of 56 vs. 47) and more likely to reside in the Phoenix Metro area (83.3% vs. 25%), provide an e-mail address (100% vs. 75%), and use antidepressants (33.3% vs. 25%). No participants were classified as depressed, and only 1 TIA participant was discharged with mild confusion.

Over the 4-week period, survey completion rates and hourly GPS location streaming for both TIA and ischemic stroke populations exceeded 75%, with lower survey completion and hourly location data rates in some weeks (Table 3). Passive data collection of survey response time, potentially an indicator of cognitive processing speed, showed the longest response times observed during the first week of the study. The volume of data collected from smartphone accelerometer sensors, activated by movements such as walking with a phone in one’s pocket, is known to vary by device; however, comparable volumes of data streamed from both cohorts across all weeks.

In exit interviews, three participants expressed that the app drained their phone battery and required them to charge their phones more frequently. Two participants expressed that they would prefer the app save their password, eliminating the need to recall passwords to complete surveys. Five participants noted no issues with the app or surveys. Among participants who were lost to follow-up (n = 4), one participant noted that she shared her phone with her husband, another mentioned that his wife was concerned about data privacy, and one expressed that the app was not user-friendly.

## 4. Conclusions and Future Work

Assessing the feasibility of using sensor-based smartphone data for digital phenotype analysis, the capture of both active and passive data appears to be feasible in both ischemic stroke and TIA populations. Active data collection for the 4-week period yielded high survey response rates (>75%) for both populations, and passive GPS data was streamed during most hours (>80%) throughout the study. Regarding accelerometer data, the large volumes of data generated suggest participants are using and carrying their devices throughout the day. Relatedly, the large standard deviation observed for TIA participants in week 4 may indicate that a participant has stopped ambulating or has left his device unattended; as such, researchers should prepare to investigate changes in behavior by scanning other passive data streams, such as GPS location.

Considering the preliminary feasibility results presented, we highlight below considerations for future researchers based on the re-design and testing stages we iterated through to inform feasible protocol design to assess the predictive potential of digital phenotyping for PSD. 

### 4.1. Participant Device Ownership and Sharing

Unless otherwise specified, a participant’s sensor data are only valid if they are generated by said participant, notably in longitudinal studies requiring baseline data for comparison. To guarantee the veracity of data collected from a patient’s device(s), it is necessary to appraise an eligible patient’s device ownership or sharing patterns prior to enrollment [30]. For example, in studies requiring participants to record passive or active data with smartphone sensors, researchers should ask eligible participants whether they share a smartphone with a caregiver, spouse, child, or other individual. While multiple instruments assessing smartphone use frequency exist, no comprehensive data about older adults, who are amongst the most likely to suffer stroke or TIA, and smartphone sharing is currently available; however, in our study, we identified smartphone-sharing patterns through dialogue with screened participants. Additionally, while older adults—or those with the functional impairment characteristic of CeVD—may receive assistance from an individual to complete survey instruments after an emergency event, it is essential to identify whether a study requires that all active and passive data be exclusively generated by the enrolled participant, i.e., a study only recording survey data through a web browser form may be able to be completed by a caregiver without compromising study data [31]. 

In contrast, the opposite may hold for a study quantifying passive data, such as smartphone keyboard screen typing patterns. While rare, malicious actors can potentially use a shared device—including public devices, like library laptops or tablets, or stolen personal devices—and input fraudulent information. Researchers should encourage participants to swiftly report theft or suspicious activity related to devices involved in a study. Additionally, as older adults, the population with highest TIA prevalence, are at a heightened risk of undetected cybercrime; researchers should routinely audit collected study data and proactively investigate any aberrations [32].

### 4.2. Participant Wireless Network and Technology Access Proficiency

Unlike physiologic biomarkers, such as gait, that require near-continuous measurements and generate large volumes of data, monitoring of behavioral symptoms, such as depressed mood, are customarily assessed via validated survey instruments. As such, researchers investigating behavior should consider generational trends and assessment time points inclusive of their target population’s digital engagement level. 

Also, the ability to successfully enter or troubleshoot log-in credentials, whether for wireless internet connectivity outside clinical settings or to access a HIPAA-compliant smartphone app to submit data, is often integral to smartphone study completion. Although eligible participants may self-report sufficient computer, tablet, or smartphone use at an eligibility screening, such assessments may be biased. In our study, some patients who indicated moderate smartphone use struggled to enter log-in credentials for the app. The Wireless Network Proficiency Questionnaire may be one tool capable of predicting Wi-Fi credential recall in aging adults or those affected with cognitive or memory impairments, like many stroke patients, making it a potential criterion for inclusion in some populations [33]. 

After a participant enrolls in a smartphone study, tech support needs over time should be expected. Specific to our study, we provided phone and e-mail support with a <24 h turnaround time to resolve wireless and cellular connectivity problems, password resets, and other issues. Though a study may be designed as fully remote, researchers whose studies involve complex devices should be prepared to offer technical support in person if required, as unique dropout reasons may drive the loss of participants. For instance, forgetting log-in credentials and technical processes associated with completing a study or the location of a cell phone charger could be early signs of cerebrovascular changes, including but not limited to vascular dementia, and could be critical to assessing health outcomes and stratifying patient cohorts [34,35,36]. Alternatively, such symptoms may be more prevalent in older adults with low socioeconomic status and limited education [37]. 

### 4.3. Active Data Capture Complexity 

For adults who have recently suffered a hospital admission or other acute event, researchers should consider the demand that thorough survey instruments place on a recovering patient’s cognition. 

In our study, we initially used the gold standard instrument for mood assessment in patients with cardiovascular changes: the 22-question Beck Depression Inventory-II (BDI-II) [38]. Patient (*n* = 3) willingness was high at enrollment; however, fewer surveys were conducted as time progressed, with each participant lost to follow-up. Feedback from one participant’s family included concerns about the survey length. 

Using publicly available databases that enrolled patients with demographics comparable to our population of interest, we applied machine learning techniques to find the most relevant predictor behaviors associated with PSD. We identified that behavior variables assessed with the eight-question Patient Health Questionnaire (PHQ-8) were more relevant than the 22 behaviors assessed with the BDI-II. To avoid overwhelming post-stroke and TIA patients with reading extensive survey questions, we switched to a shorter assessment instrument with an emphasis on more valuable predictors [39].

### 4.4. Passive Data Collection with a Fail-Safe Strategy 

Inherent to reproducible remote monitoring research is the need to cross-check measurements by comparing them to a reliable standard. When hands-on observation is impossible, researchers must design studies with ingrained flexibilities to indicate a failed sensor or wireless data stream. One such flexibility is the inclusion of a sleep biomarker as a reference biomarker against which to compare different attributes of other biomarkers. Its mediative influence on other biomarkers, like blood pressure and glucose levels, has been well documented and may one day be further elucidated by digital biomarker research [40,41]. Sleep will not necessarily be an appropriate endpoint for every smartphone study; however, characterizing sleep attributes using digital measurements can afford researchers a glimpse into software and hardware functions outside of a physical observation setting [42]. This is especially valuable in studies using one or more of a participant’s devices, also known as bring-your-own-device (BYOD) studies [43]. 

The high rate of hourly GPS data completeness observed in our study opens the door for analysis of correlations between (passive) GPS measurements and (active) patient-reported sleep patterns. Changes in sleep patterns by themselves may not indicate improvement or deterioration in participant status; however, comparing the precision of remote measurements to self-reported sleep patterns over time can provide insight into smartphone sensor performance. With self-reported assessments at thoughtful time intervals, the failure of a smartphone sensor tracking sleep does not necessarily render related data useless. Collecting multiple modes of data to track a single biomarker—in this case, sleep—should be considered on a case-by-case basis and may be more pertinent in older adult studies where participants may be less tech-savvy. Regardless, researchers should be mindful that sensors and data streams in BYOD studies, like ours, must be monitored carefully. 

### 4.5. Follow-up Assessment Scheduling to Accommodate Patients, Family, and Caregiver(s)

When monitoring participants after a TIA or stroke hospitalization, considerations for the availability of family members, like a spouse or a caregiver, are paramount. Furthermore, many CeVD patients are actively managing multiple health conditions at once, requiring them to visit specialists and undergo physical therapy. Such commitments are time-consuming and, in many cases, exhausting. As such, researchers studying digital phenotypes associated with cerebrovascular dysfunction should be prepared to adapt initial study designs to respond to patients’ evolving needs.

At the outset, we believed exit interview availability between standard business hours (8 AM–5 PM) would be sufficient; however, after meeting with patients, it was clear that follow-up assessments would need to be scheduled around family availability if a patient’s condition worsens or technical troubleshooting assistance with teleconference software is needed [44]. Given that minority patients are poorly represented in digital health studies, an accommodating follow-up assessment strategy must be employed to prevent the exacerbation of health disparities in the digital realm. 

Specific to TIA patients, we observed that some patients delay seeking help by multiple days due to the condition’s transient nature. As such, enrolling and assessing patients immediately after a TIA event is not always feasible. We adapted our study to no longer consider the first 72 h after hospital admission as a critical post-TIA monitoring period, instead reducing the number of assessments required of participants to resemble the first +/−7 days after a TIA more accurately. 

### 4.6. Embedded Study Designs

To date, most digital phenotyping studies have been observational studies to evaluate the feasibility of using an early-stage technology [45]. Numerous studies have been published assessing biomarkers linked with quality of life or behavioral changes in patients with a wide range of diagnoses [45]. These studies typically enroll fewer than 100 participants, rendering the findings of such investigations limited regarding generalizability [46]. 

At first, our study design focused exclusively on a frequently overlooked patient population: TIA patients [47]. These patients suffer from temporary symptoms and, as such, are subject to less intensive monitoring than patients with other cerebrovascular disease (CeVD) conditions, such as ischemic stroke. After meeting with eligible participants or filtering those diagnosed with TIA but ineligible for our study, we recognized the need to pivot quickly to meet our target enrollment numbers. The decision was made to modify the inclusion criteria to incorporate post-stroke patients, who are also at a heightened risk of future stroke, as a reference cohort for our TIA cohort. Additionally, recruiting post-stroke patients was our best option to minimize the impact of eligible older adult patients being less willing or able to participate in a DHT study. 

The recruitment of post-stroke patients represented an embedded study design within our institution’s pre-existing stroke risk monitoring program [48]. Such a move can potentially improve retention and enhance care, as patients are already engaged with an institution for follow-up visits. 

### 4.7. Mindful of Data’s Role in Multimodal Studies

As the popularity of wearable devices continues to rise, the volume of data collected outside clinical settings will grow exponentially; yet, these data are less useful than blood biomarkers and gold-standard assessments concerning diagnoses and prognoses [49,50]. Unraveling the complexity of phenotypes associated with human disease can be aided with real-time, digital data; however, the value of data, such as digital phenotyping measurements, is tied to their ability to augment rather than replace multimodal clinical data sources, such as EHRs, imaging, and wearable devices [51]. The use of smartphones in multimodal studies can offer a more accurate and comprehensive understanding of patient conditions, allowing for tailored therapeutic approaches [52]. This is especially relevant in the context of neurological disorders, where the nuances of the disease can be better understood and managed via detailed, real-time monitoring provided by DHTs [52]. 

## Figures and Tables

**Table 1:** Hypotheses for Primary Smartphone Sensor Active and Passive Features.

Active Data Feature	Feasibility Assessment—Summary Metric	Future Clinical Use—Intra-Individual Metric
Mood Survey	Survey completion and submission rate (%)	Survey scores will increase from controls to PSD
**Passive Data Feature**	**Feasibility Assessment—Summary Metric**	**Future Clinical Use—Intra-Individual Metric**
Mood Survey Response Time	Survey completion time recorded (mean, standard deviation)	Survey completion time will increase from controls to PSD
Activity (Accelerometer Quantified)	Average of weekly sum of accelerometer volume generated (mean, standard deviation)	Average weekly activity will decline from controls to PSD
Social Engagement (GPS Quantified)	Hours with GPS location data per week (%)	Average number of trips outside the home will decline from controls to PSD

**Table 2:** Baseline Characteristics.

Characteristics	Participants	
	Ischemic Stroke (*n* = 4)	TIA (*n* = 12)
Age (years), mean (SD)	47 (14.9)	56 (18.1)
Age (years): min-max	25–56	23–81
Male (*n*)	3	7
Ethnicity (*n*)		
White	3	9
Hispanic	0	1
Native American	1	0
Asian	0	1
Black	0	0
Unknown	0	1
Use of phone (*n*)	4	12
Use of e-mail (*n*)	3	12
Resides in Phoenix Metro Area (*n*)	1	10
Mood Status at Discharge (*n*)		
Normal	3	11
Depressed	0	0
Missing	1	1
Cognitive Status at Discharge (*n*)		
Normal	3	8
Confused	0	1
Missing	1	3
Antidepressant Use? (*n*)	1	4

**Table 3:** Summary Metrics for Passive and Active Smartphone Sensor Data.

		Participants	
Data Feature	Feasibility Assessment—Summary Metric	Ischemic Stroke (*n* = 3)	TIA (*n* = 8)
Mood Survey (Active)	Survey completion and submission rate (%)		
	Week 1	66.7	75.0
	Week 2	100.0	75.0
	Week 3	100.0	87.5
	Week 4	66.7	75.0
Mood Survey Response Time (Passive)	Survey completion time recorded (mean seconds, standard deviation)		
	Week 1	182.7 (175.6)	75.6 (51.1)
	Week 2	50.8 (27.7)	63.9 (54.7)
	Week 3	41.3 (15.2)	42.5 (25.7)
	Week 4	31.4 (17.5)	72.9 (38.2)
Activity (Passive)	Average of weekly sum of accelerometer volume generated (mean bytes, standard deviation)		
	Week 1	23,031,109.0 (10,838,334.6)	30,837,116.7 (29,557,213.9)
	Week 2	19,975,074.2 (12,015,996.8)	29,580,727.3 (21,580,446.0)
	Week 3	18,130,797.6 (7,425,125.0)	27,469,212.8 (26,079,380.8)
	Week 4	27,868,016.3 (11,006,512.0)	25,536,878.1 (22,581,041.5)
Social Engagement (Passive)	Hours with GPS location data per week (%)		
	Week 1	81.2	77.9
	Week 2	79.0	91.6
	Week 3	100.0	81.3
	Week 4	99.0	85.6

## Data Availability

The original contributions presented in this perspective study are included in the article. Further inquiries can be directed to the corresponding author.
